# Notch signalling in healthy and diseased vasculature

**DOI:** 10.1098/rsob.220004

**Published:** 2022-04-27

**Authors:** Francesca Del Gaudio, Dongli Liu, Urban Lendahl

**Affiliations:** ^1^ Department of Cell and Molecular Biology, Karolinska Institutet, 171 77 Stockholm, Sweden; ^2^ Department of Pediatrics, The First Affiliated Hospital of Guangxi Medical University, Nanning 530021, People's Republic of China

**Keywords:** Notch signalling, vasculature, endothelial cell, pericyte, vascular smooth muscle cell, cerebral small vessel disease

## Abstract

Notch signalling is an evolutionarily highly conserved signalling mechanism governing differentiation and regulating homeostasis in many tissues. In this review, we discuss recent advances in our understanding of the roles that Notch signalling plays in the vasculature. We describe how Notch signalling regulates different steps during the genesis and remodelling of blood vessels (vasculogenesis and angiogenesis), including critical roles in assigning arterial and venous identities to the emerging blood vessels and regulation of their branching. We then proceed to discuss how experimental perturbation of Notch signalling in the vasculature later in development affects vascular homeostasis. In this review, we also describe how dysregulated Notch signalling, as a consequence of direct mutations of genes in the Notch pathway or aberrant Notch signalling output, contributes to various types of vascular disease, including CADASIL, Snedden syndrome and pulmonary arterial hypertension. Finally, we point out some of the current knowledge gaps and identify remaining challenges in understanding the role of Notch in the vasculature, which need to be addressed to pave the way for Notch-based therapies to cure or ameliorate vascular disease.

## Graphical abstract

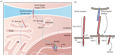

## 1. Introduction

The vasculature is composed of blood vessels—ranging in size from the large aorta to the ultrathin capillaries—that secure the transport of oxygen and nutrients to all parts of the body. From the discoveries by William Harvey in the 1600s of the circulatory vascular system and its two major branches (the systemic and pulmonary circulation), we have been intrigued by the complexity of the vasculature, and there has been a continuous quest to unravel the mechanisms that generate and maintain our blood vessels. Progress was first made at the anatomical level, leading to a detailed characterization of the vasculature down to its finest capillary branches [1]. With the advent of more advanced light microscopy methods, the different cell types that make up the blood vessels—endothelial and mural cells—could be identified. More recently, in the molecular era, we have begun to gain insights into the molecular mechanisms that govern vascular development, such as cellular proliferation and differentiation, the molecular portraits of the various cell types and mechanisms for branching of the vasculature [1]. These insights into the development and homeostasis of the normal vasculature were accompanied by an increased understanding of diseases that affect the blood vessels, ranging from stroke, hypertension and atherosclerosis to genetic diseases perturbing specific aspects of the vasculature. An important, but possibly unsurprising, lesson from the molecular endeavours to decode the vasculature is that many of the most highly conserved signalling mechanisms that are important for cellular differentiation and homeostasis across different organs of the body also are at work in building and maintaining the vasculature. This cadre of signalling mechanisms includes Shh, Wnt, JAK/STAT, YAP/TAZ, BMP/TGFb and, last but not least, Notch—the main focus of this review.

## 2. The Notch signalling pathway

**Figure 1.**
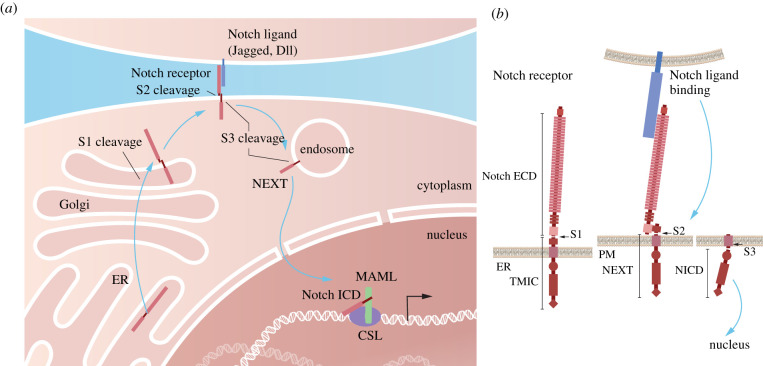
The Notch signalling pathway. (*a*) Schematic depiction of the major steps in the Notch processor maturation and routing in the cell. The Notch receptor is produced in the endoplasmic reticulum (ER) and the first proteolytic cleavage (S1) takes place in the Golgi compartment. After ligand interaction at the cell surface, S2 cleavage occurs. The Notch receptor subsequently undergoes S3 cleavage, at the cell surface or frequently in the endosome, and the resulting intracellular domain (ICD) is transported to the nucleus, where it becomes part of the ternary ICD/CSL/MAML transcriptional complex, which regulates downstream gene activation. (*b*) Schematic presentation of the proteolytic processing steps for the Notch receptor. The Notch receptor undergoes S1 cleavage in the heterodimerization domain (HD). S1 cleavage generates the extracellular domain (ECD) and the transmembrane/intracellular fragment (TMIC) moieties. The ECD is liberated into the extracellular space or taken up by the ligand-presenting cell, while the TMIC moiety is cleaved by S2 cleavage to form the Notch extracellular truncated (NEXT) domain. Finally, S3 cleavage in the membrane liberates the ICD (NICD), which is transported to the nucleus. PM, plasma membrane.

The Notch signalling pathway operates in most, if not all, multicellular species, and controls cell differentiation through cell–cell communication. The molecular mechanism for transmitting the Notch signal from the cell surface to the nucleus has been extensively reviewed [2–4] and will, therefore, be only briefly summarized here. Notch receptors (Notch1–4) are large transmembrane receptors that undergo a series of proteolytic cleavages. The first cleavage (S1 cleavage) occurs in the Golgi compartment by furin-like convertase. The bipartite receptor is next presented at the cell surface, where it can interact with ligands (Jagged (Jag) 1 and 2 or Deltalike (Dll) 1,3 and 4), which are transmembrane proteins presented at juxtaposed cells (figure 1). The ligand–receptor interaction causes a conformational change in the so-called negative regulatory region (NRR) of the Notch receptor, which exposes a cleavage site for disintegrin and metalloproteinase 10 (ADAM10). The resulting S2 cleavage splits off the extracellular domain of the receptor and leaves a Notch extracellular truncation (NEXT) moiety in the membrane. The NEXT moiety is subsequently cleaved by the γ-secretase complex (S3 cleavage), which results in the release of the intracellular domain (Notch ICD) that via the endosomal route travels to the cell nucleus (figure 1). In the nucleus, Notch ICD forms a ternary complex with the DNA-binding protein CSL (also known as RBP-J or CBF1) and Mastermind-like (Maml). Notch ICD converts CSL from a repressor and the Notch ICD/Maml/CSL ternary complex activates expression from downstream genes in the Notch pathway, including Hes and Hey genes (figure 1). Notch receptors are also subject to a variety of post-translational modifications that modify signalling output, including glycosylation, phosphorylation, hydroxylation, ubiquitylation and sumoylation [5]. In addition to the ‘canonical' form of signalling, there are also several non-canonical modes of Notch signalling, which, however, are mechanistically less well understood [6]. While the architecture of the Notch pathway is relatively simple—there are, for example, no kinase amplification steps in the core pathway—signalling output is versatile [7]. Notch signalling operates in a large variety of cell and organ contexts, providing different molecular outputs appropriate for each cell type. How this diversity is generated is still rather enigmatic and an area of active research.

## 3. The vasculature

**Figure 2.**
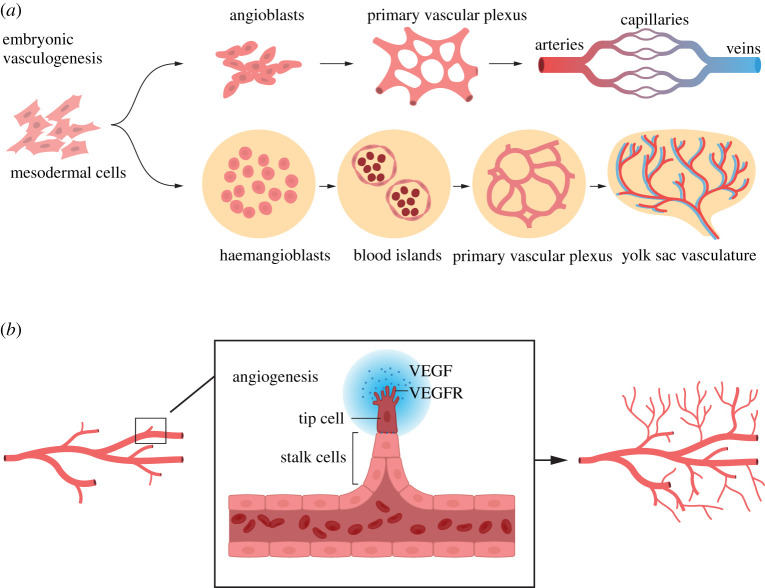
Vasculogenesis and angiogenesis. (*a*) Vasculogenesis: the embryonic and extraembryonic vasculature originates from mesodermal cells. During the embryonic vasculogenesis, mesodermal cells differentiate to angioblasts, which form a primary vascular plexus. The blood vessels in the vascular plexus subsequently acquire arterial (red) and venous (blue) identities along an arterial–capillary–venous axis. During extraembryonic (yolk sac) vasculogenesis blood islands are initially formed from haemangioblasts, and the blood islands later generate a primary vascular plexus. This extraembryonic vascular plexus subsequently produces the vasculature in the yolk sac. (*b*) Angiogenesis: from an initial vascular tree, new blood vessels are generated by sprouting. Sprouting occurs by a leading tip cell, which responds to vascular endothelial growth factor (VEGF) via VEGF receptors (VEGFRs) and which is followed by stalk cells that form the lumen of the new sprouting vessel.

The vascular system originates through a process called vasculogenesis, which is characterized by de novo formation of blood vessels early in embryogenesis. Derived from mesodermal cells, endothelial progenitor cells (angioblasts) form a primitive vascular network (a plexus) starting around embryonic day (E) 7.5 in the mouse embryo. The early coalesced endothelial cells (ECs) become coated with mural cells (see below) and undergo a subsequent remodelling and differentiation into arteries, veins and capillaries [8] (figure 2*a*). In parallel with the initial formation of a vasculature in the embryo proper, extraembryonic vasculogenesis occurs in the yolk sac, where the vasculature is built from ECs derived from haemangioblasts, which also give rise to haematopoietic cells [9]. The haemangioblasts cluster into blood islands with ECs at the perimeter and primitive haematopoietic cells in the centre (figure 2*a*).

Vasculogenesis is followed by a process called angiogenesis, which is defined as the generation of new blood vessels from the basic vascular network laid down during vasculogenesis. An important aspect of angiogenesis is the sprouting of new vessels from pre-existing ones. In the early vasculature, sprouting is executed by so-called tip cells, which take the lead in growing the new vessels and which are followed by stalk cells that form the lumen of the new vessel [10] (figure 2*b*).

**Figure 3.**
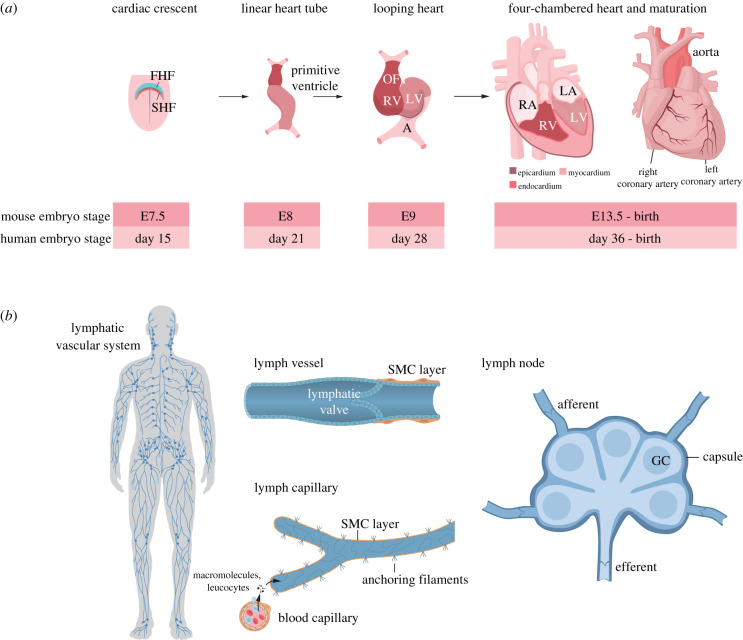
Development of the heart and lymphatic system. (*a*) The heart originates from the first and second heart fields (FHF and SHF, respectively) in the early embryonic mesoderm. From a linear heart tube containing a primitive ventricle and atrium, a looping heart with an atrium (A), left and right ventricles (LV, RV) and an outflow tract (OFT) is formed. The mature heart contains a left and right atrium (LA, RA) as well as the left and right ventricles (LV, RV). The locations of the epicardium, myocardium and endocardium as well as the right and left coronary arteries are depicted in the figure, along with a time axis for mouse and human heart development. (*b*) The lymphatic system extends across the human body (left). It is composed of lymphatic vessels with lymphatic valves and zipper-like junctions, which are coated by smooth muscle cells (SMCs) (upper middle). A lymphatic capillary and a blood vessel capillary are depicted, with macromolecules and leucocytes traversing from the blood vessel to the lymphatic capillary (lower middle). To the right, a lymph node composed of an inner medulla with germinal centres and an outer cortex is shown. Lymph enters into the lymph node from afferent lymph vessels and exits via efferent lymph vessels. GC, Golgi compartment.

A central component of the vascular system is the heart, which is the first functional organ to form in the embryo [11]. Cardiogenesis starts at E7.5 when mesodermal cells from the cardiac crescent coalesce into a primitive heart tube (figure 3*a*). The heart is formed from two principal founding cell populations: the first (primary) heart field, which gives rise to both atria and the left ventricle, and the second heart field, which generates the right ventricle and the outflow tract. The early heart is a three-layered structure, composed of an inner endocardium, a myocardium in the middle and an outer epicardium. Branching from the aorta, the coronary arteries wrap around the heart and provide the heart with blood circulation (figure 3*a*).

In addition to the blood-transporting vasculature, the lymphatic vasculature drains plasma and proteins that are extravasated from the interstitium (figure 3*b*). The uptake of fluid into the lymphatic vessels is conducted via permeable junctions in the lymphatic ECs [12], and the fluid is later transported back to the blood circulation. The lymphatic system plays an important role in immune surveillance, by importing antigens and exposing them to antigen-presenting cells in the lymph nodes. Lymphatic vessels are distinct from blood vessels in several ways, for example by the lack of pericytes (see below) around lymphatic capillaries.

**Figure 4.**
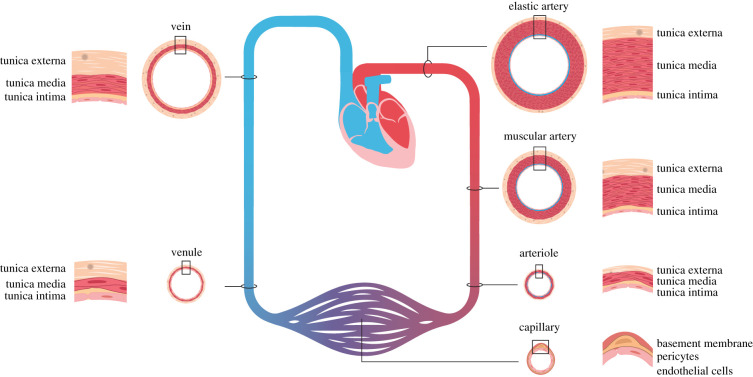
Cellular organization of different types of blood vessels. Blood vessels of different sizes, ranging from elastic (e.g. aorta and pulmonary artery) and muscular (e.g. femoral and radial artery) arteries via arterioles to capillaries and further on to venules and veins, are depicted. The cellular organization of endothelial and mural cells (VSMCs and pericytes) of the differently sized vessels is shown in cross-section. Note that muscular arteries contain more VSMCs in the tunica media than elastic arteries. Arterioles have a VSMC coat but largely lack elastic tissue. Capillaries have a single EC layer and are covered by pericytes rather than VSMCs. Venules and veins are thin walled and less elastic than arteries.

At the cellular level, the vasculature contains ECs that form the inner layer, which is surrounded by mural cells: vascular smooth muscle cells (VSMCs) and pericytes. VSMCs cover larger vessels, i.e. elastic and muscular arteries, arterioles and veins, while pericytes cover the thinnest vessels, i.e. the capillaries and venules (figure 4). The density of mural cell coverage varies, with an almost complete VSMC coverage of elastic and muscular arteries, a more moderate VSMC coverage of veins and a sparse layer of pericytes covering the capillaries. Smaller vessels have a single-layer VSMC coat whereas elastic arteries, such as the aorta, contain a more complex coating with a tunica intima, a tunica media, harbouring up to six layers of VSMCs, and an outer tunica externa (figure 4).

In addition to serving as a conduit system for nutrients, oxygen and waste products, the vasculature plays an important role in maintaining blood pressure through the regulation of vascular tone, which is achieved by a balance between vasoconstrictor and vasodilator signals. The vascular endothelium is able to sense haemodynamic changes and to respond via the release of vasoconstrictors and vasodilators [13]. VSMCs regulate vascular tone through expressing proteins critical for contraction or dilation. Under pathological conditions, VSMCs lose their contractility and switch to a synthetic phenotype, which is characterized by increased proliferation, migration and extracellular matrix synthesis, contributing to the occlusion of blood vessels and increased vascular resistance [14,15]. The VSMC phenotype switching is in part regulated by vasoactive substances, such as endothelin, which are produced by ECs.

## 4. Notch and vascular development

### 4.1. Vasculogenesis

Notch signalling is important from the onset of vasculogenesis, as witnessed by early embryonic vascular phenotypes in mice where genes in the Notch pathway have been genetically ablated. Thus, targeting of Hey1 and Hes1 [16], Notch1 and Notch4 [17] and Jagged1 [18] results in a disorganized embryonic vascular bed. Similarly, restricting targeting of Notch1 or CSL to ECs resulted in dramatic vascular phenotypes [17,19,20], indicating that an endothelium-specific function of Notch is required for proper organization of the embryonic vascular plexus. The yolk sac vasculature is also affected by reduced Notch signalling as ablation of Dll4 resulted in an aberrant vasculature in the yolk sac [19,21,22].

Notch gene targeting experiments furthermore revealed a role for Notch signalling in arterial versus venous fate specification: loss of Notch signalling led to reduced arterial endothelial differentiation at the expense of enhanced venous endothelial differentiation, and considerable progress has been made in decoding the underpinning molecular principles. Arterialization is mediated by Notch signalling acting epistatically over the Eph/ephrin signalling pathway, which is an important regulator of vascular cell fate specification [23]. Specifically, Notch activates ephrinB2 and simultaneously represses EphB4 in the early vasculature, leading to the acquisition of an arterial endothelial fate [20,24–26]. Upstream of Notch, vascular endothelial growth factor (VEGF) plays a central role by upregulating Notch1 and Dll4 at the prospective arterial side of the vasculature. At the venous side, suppression of Notch by COUP-TFII promotes the venous fate in ECs [27], although a direct link between COUP-TFII and Notch was challenged by a more recent study [28]. In zebrafish, it has been demonstrated that Notch acts early, in seemingly uncommitted progenitors, to control the arterial versus venous differentiation [29]. More mechanistic insights into the VEGF–Notch–Eph/ephrin axis for arterial specification have been gained in recent years. It has been shown that tetraspanin 18 regulates both Notch and VEGF signalling during developmental angiogenesis [30]. Benedito and co-workers [31] demonstrated that elevated VEGF and Notch levels suppress the cell cycle and promote enrolment of ECs into arteries. Blocking the cell cycle through ablation of Myc promoted the arterial fate choice independently of Notch, suggesting that a key role for Notch in the arterialization process is to dampen cell cycle activity.

Although the initial steps of arterial versus venous differentiation occur prior to differences in blood flow between arteries and veins have been established, it has been extensively discussed whether vascular flow (haemodynamics) is important for Notch signalling and that a higher pressure at the arterial side may contribute to arterial specification. Shear stress indeed upregulates vascular Notch signalling [32,33], suggesting a potential role for flow in modulating Notch signalling strength. Gerhardt and co-workers [29], however, demonstrated that Notch acts prior to the onset of flow in regulating arterial versus venous fates in zebrafish. It was also recently shown that haemodynamic shear stress plays a role in endothelial barrier function, and this at least in part is mediated via Notch signalling [34]. This was, however, proposed to be executed via the transmembrane portion of Notch1, which would represent a novel non-canonical mode of signalling and the mechanistic basis for this requires further research.

Lymphatic vessels are not formed through vasculogenesis followed by angiogenesis. Instead, lymphatic ECs are generated from venous ECs during embryogenesis [12]. This transition towards a lymphatic endothelial fate is regulated by transcription factors such as Sox18, Prox1 and COUP-TFII and is under the influence of VEGF signalling (VEGFR-3 and VEGF-C). EphrinB2 is important for lymphatic sprouting [12], and a recent report unveils a specific role for Notch4 in the regulation of vessel diameter in the developing lymphatic plexus [35]. During the postnatal stages, Notch signalling is also important for the lymphatic system [36], and Notch serves a role in restricting VEGF-induced lymphatic endothelial sprouting [37]. The role of Notch in maintaining lymphatic EC junctional integrity has also been described [38,39].

### 4.2. Angiogenesis

During angiogenesis, new blood vessels sprout from the vessels in the existing initial plexus, a process mediated by tip and stalk cells in the growing vasculature (figure 2*b*). Notch signalling and VEGF are important arbiters of the tip/stalk specification process. Sprouting is initiated by elevated VEGF levels (figure 2*b*), which activate the ERK pathway and upregulate the expression of Dll4 in the prospective tip cells [40]. Integrin signalling, via laminins a2b1 and a6b1, has also been shown to upregulate Dll4 in EC [41]. The elevated Dll4 levels activate Notch receptors in the neighbouring prospective stalk cells, leading to downregulation of VEGFR2 and VEGFR3 expression and stabilization of the stalk cell fate [42,43]. Dll4 furthermore acts *cis*-inhibitory for Notch receptors in the tip cells, which reduces Notch signalling, promoting a higher level of ERK signalling in the tip cells [44,45]. Jagged1 is expressed in the stalk cells, but is post-translationally modified by Fringe proteins, and thus is not a good inducer of Notch receptors in *trans*, i.e. on the tip cells [46]; for a review see [47]. A role for YAP/TAZ signalling in angiogenesis was unveiled that has a bearing on Notch signalling: when YAP/TAZ signalling was decreased through targeting of LPA4/LPA6, this led to elevated Dll4 expression and impaired sprouting [48]. The chemokine receptor CXCR4 has also been invoked in EC sprouting, and in zebrafish cxcr4a is regulated by Notch; initially, cxcr4a is induced by Notch, but is later instead reduced by Notch, thus preventing blood vessel hypersprouting [49]. In mice, it was demonstrated that CXCR4, together with VEGF-A, enhances endothelial sprouting and proliferation in a Notch-dependent manner [50]. Notch signalling also plays a role in regulating EC junctions via modulation of vascular endothelial (VE) cadherin levels [51]. The function of Notch signalling in biasing tip and stalk cells is a good example of lateral inhibition, a mode of differentiation frequently employed in, for example, *Drosophila melanogaster* or *Caenorhabditis elegans*. During lateral inhibition, high levels of Notch ligand expression in one cell in a relatively homogeneous cell population activate Notch signalling in the neighbouring cells, forcing them to not take on the same cell fate as the cell with high ligand expression but opt for an alternative differentiation fate; for a review see [52].

While the role of Notch signalling in the tip/stalk segregation process appears to be general, the consequences of perturbing endothelial Notch signalling differ between different organs (see [9] for a review). Interestingly, targeting of Notch1 in ECs in a wild-type or a Notch4^−/−^ background resulted in effects in the vasculature of long bones but not in the intestine or retina [53]. In the coronary vasculature, endothelial targeting of Notch1 on a Notch4^−/−^ background or targeting of ADAM10 (which executes S2 cleavage of the Notch receptor) led to enlarged subepicardial vessels and loss of mural cell ensheathment [53–55]. Hypomorphic Notch2 affected the formation of specific vascular beds such as the hyaloid vasculature of the eye, the glomerular capillary tuft and the myocardium [56]. Endothelial inactivation of Notch1 or CSL resulted in enlarged malformed vessels in the liver [57], and Dll4 haploinsufficiency led to a dysmorphic microvasculature in the lung [58].

### 4.3. Mural cell differentiation

The initial endothelial vasculature needs to be coated with mural cells (VSMCs and pericytes), and both platelet-derived growth factor (PDGF) and Notch signalling are important for mural cell coating. The PDGF signalling pathway plays an important role in the differentiation, proliferation and recruitment of mural cells to the endothelium. The PDGFB ligand is produced and secreted from the endothelium, promotes mural cell differentiation from mesenchymal progenitor cells and serves as an attractant for mural cell investment on the blood vessels [59,60]. Mural cells express the PDGF receptor beta (PDGFRB) and perturbation of the PDGF signalling pathway results in loss of pericytes and VSMCs, manifested, for example, in a leaky blood–brain barrier [61]. Notch signalling plays a key role at the earliest stage of pericyte development from mesenchymal cells in zebrafish [62]. At later stages, the role of Notch signalling, in particular of Notch3, the predominant Notch receptor in mural cells, in pericyte function is less clear. Notably, there are conflicting views on whether pericytes are affected in Notch3^−/−^ mice and in patients with cerebral autosomal dominant arteriopathy with subcortical infarcts and leucoencephalopathy (CADASIL), which is caused by NOTCH3 mutations (see below) [63–65]. In zebrafish, Notch3 function, however, seems necessary for pericyte generation [66].

Little is known about the role of Notch signalling at the earliest steps of VSMC differentiation from mesenchymal progenitor cells, but Notch3 is critically important for VSMC maintenance, as VSMCs are progressively lost at later postnatal stages in Notch3^−/−^ mice [63,67]. In an important study by Joutel and co-workers [68], a shift from an arterial towards a more venous VSMC fate was noted in Notch3^−/−^ mice (see below for details). In subsequent studies, it was observed that Notch signalling executes a VSMC function, in part via regulation of PDGF signalling: Notch3 promotes PDGFRB expression and PDGFRB mRNA levels were reduced in the Notch3^−/−^ mice [69]. The discovery that Notch3 regulates PDGFRB links two important signalling pathways in VSMC differentiation and a Notch–PDGF signalling axis may in fact not be restricted only to mural cells, as it was recently observed that a NOTCH3 gain-of-function (NOTCH3^L1519P^) elevated PDGFRB expression in infantile myofibromatosis, a non-metastatic cancer formed in bone, skin and muscle [70]. Progress is also made in understanding the mechanistic details of Notch3 function in VSMCs, and the basement membrane protein Nidogen-2 was recently found to interact with Jagged1 and stabilize the Jagged1–Notch3 interaction [71].

## 5. Notch signalling is important for vascular homeostasis

In addition to pivotal roles in the development of the vasculature, vascular Notch signalling is important at later stages, and it is increasingly realized that vascular Notch signalling plays a role as a regulator of homeostasis in the postnatal vasculature. As discussed above, ablation of CSL or Notch1 in postnatal ECs leads to enlarged vessels, dilated sinusoids and disorganized vascular architecture in the liver, accompanied by reduced levels of ephrin B2 [57]. Analysis of kidneys from mice with an endothelial-specific ablation of the ADAM10 gene (which regulates S2 cleavage of Notch receptors; see above) revealed the persistence of diaphragms in the fenestrated kidney ECs, enlargement of vessel diameter in the kidney glomeruli and an increase in intussusception events in capillary loop formation in the glomeruli, collectively suggesting a delay in maturation of the kidney vasculature [72]. Endothelial targeting of ADAM10 was shown to be important for decidual angiogenesis during the initiation of pregnancy and leads to reduced fertility in mice [73].

Perturbed vascular Notch signalling can also have ‘spillover’ consequences for the homeostasis of surrounding organs and non-vascular cell types. Notably, Dll4 expression on sinusoidal ECs plays a role in the differentiation of Kupffer cells (resident liver macrophages) [74]. Ablation of Dll1 in ECs in spleen and bone marrow resulted in abrogation of a specific monocyte subpopulation (Ly6C^lo^ monocytes) [75]. Postnatal endothelial deletion of CSL not only decreased the number of vessels and ECs in long bones, but also caused a shortening of long bones and loss of bone mass [76]. Fischer and co-workers [77] provided interesting insights into a role for endothelial Notch signalling in regulating metabolism in the heart and skeletal muscle. Analysis of the heart in mice with endothelial CSL targeting or perturbed Dll4 function showed that fatty acid transport across the ECs into the heart was inhibited, leading to a switch in metabolic regulation of cardiomyocytes towards higher glucose uptake and cardiac hypertrophy [77]. In a subsequent study, mice with enhanced endothelial Notch signalling in ECs (by activating Notch1 ICD specifically in ECs) showed a dampened reduction of blood sugar levels in response to insulin [78], indicating that insulin transport across the endothelium to the muscles was impaired in response to elevated Notch signalling. These examples show that perturbing Notch signalling in the endothelium not only impacts the vascular system *per se* but also has dramatic effects on other cell types and important physiological processes in various organs. To molecularly decode such ‘beyond vasculature' effects of vascular Notch perturbation is important, not least as a basis for future therapy development, as the effects relate to processes that are central from a disease perspective, such as metabolism and immune regulation.

As discussed in the previous section, under pathological or stress situations, VSMCs may switch from a contractile to a synthetic phenotype, and several lines of evidence support the notion that Notch signalling is an important regulator of the balance between the contractile and synthetic phenotypes (see [79] for a review). Notch2, although not present in arterial VSMCs during vascular homeostasis, was reported to be expressed in non-proliferating regions of injured vessels, and mediate proliferation of medial VSMCs through cell cycle arrest [80]. Overexpression of Notch1 or Notch3 ICD in VSMCs resulted in downregulation of contractile markers such as myosin, actin and smoothelin [81], induced VSMC proliferation [82] and abrogated apoptosis [83]. A number of *in vitro* studies corroborate that Notch signalling promotes the synthetic phenotype [84,85], although with the potential caveat that some of these studies were conducted in C3H10T1/2 cells rather than in VSMCs. The current view is thus that high levels of Notch activation may tilt VSMCs towards the synthetic phenotype, but a deeper analysis of how Notch regulates VSMC homeostasis and phenotypic plasticity, especially *in vivo*, is warranted, in particular as one report identified a VSMC switch towards a synthetic phenotype in coronary vessels from Notch3^−/−^ mice [86]. In addition to serving as a regulator of VSMC phenotype switching, Notch signalling regulates vascular tone. Notch3^−/−^ mice showed aberrant vascular tone regulation in distal resistance arteries (such as the brain and tail arteries), but not in large arteries (such as carotid arteries) [87]. Similarly, Notch3^−/−^ mice were largely insensitive to vasoconstrictors and vasodilators, suggesting that Notch3 is critically required for the adaptive vasoactive response [88].

The role of Notch for pericyte maintenance during vascular homeostasis is currently more enigmatic. One report observed that pericyte numbers were relatively unaffected in the Notch3^−/−^ mouse, while there was a considerable loss of VSMCs [63]. By contrast, another report described pericyte loss in the retina of diabetic Notch3^−/−^ mice [64]. Similarly, in CADASIL, which is caused by NOTCH3 mutations (see below), there are conflicting views on whether pericytes are affected or not [89,90]. More research is thus required to precisely pin down the role of Notch signalling in pericyte maintenance and homeostasis.

## 6. Notch and vascular disease

**Figure 5.**
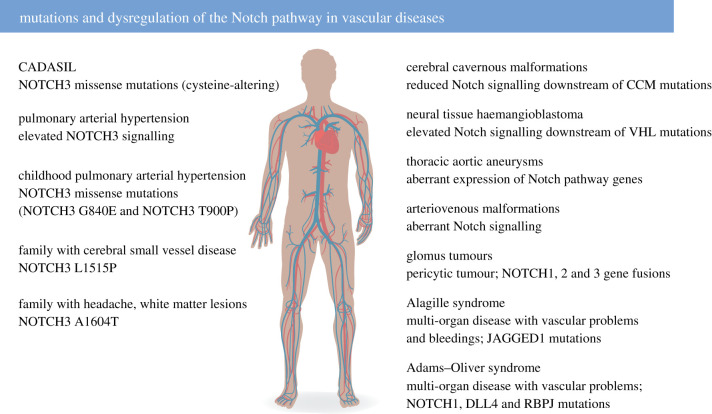
Dysregulated Notch signalling in vascular disease. Left: Diseases linked to mutations or aberrant regulation of NOTCH3. Right: Diseases linked to mutations or aberrant regulation of other genes in the Notch signalling pathway. CCM, cerebral cavernous malformation; VHL, von Hippel–Lindau.

Considering the important role of Notch signalling in vascular development and homeostasis, it is logical that dysfunctional Notch signalling has been observed in a number of vascular diseases (figure 5). Mutations causing vascular disease have been observed in genes encoding various NOTCH receptors and ligands as well as the gene encoding CSL (RBPJ). An important Notch vascular disease is CADASIL, which is caused by NOTCH3 mutations [91]. Interestingly, the NOTCH3 mutations almost exclusively affect the number of cysteine residues in the 34 epidermal growth factor (EGF)-like repeats of the NOTCH3 extracellular domain (NOTCH3 ECD) [92]. CADASIL manifests with migraine, white matter lesions, lacunar ischaemic infarcts and, importantly, degeneration of VSMCs, the primary cell type expressing NOTCH3 (for a review see [93]). CADASIL-mutated NOTCH3 ECDs accumulate outside the VSMCs in aggregates called granular osmiophilic material (GOM), which are visible by electron microscopy. The prevalence of CADASIL is 2–5 : 100 000, but it is likely to be considerably underdiagnosed [94]. An important question is whether CADASIL should be considered an aggregation disease or whether dysregulated Notch signalling is the critical pathomechanism. The prevalent view is that CADASIL, because of the NOTCH3 ECD aggregates and GOM, is an aggregation disease, similar to, for example, Parkinson's disease and Alzheimer's disease. In line with this reasoning, NOTCH3 mutations cause the formation and retention of aggregates in the endoplasmic reticulum (ER), leading to impaired VSMC proliferation [95]. Misfolding of NOTCH3 can also lead to ER stress in CADASIL VSMCs [96], and induction of Nox5 via NOTCH3 and ROCK [97]. In support of protein aggregation rather than signalling defects as a cause for CADASIL, several of the cysteine-altering NOTCH3 mutations in CADASIL are considered to be signalling neutral, i.e. not affecting the magnitude of the Notch downstream signalling output [93]. There may, however, be exceptions to this norm, as Arboleda-Velasquez and co-workers [98] identified a CADASIL mutation with hypomorphic signalling, and where the CADASIL-like phenotype in a mouse model could be partly restored by treating the mice with a NOTCH3-activating antibody (see also the Notch therapy section). Interestingly, accumulation of GOM in Notch3-deficient mice on a heterozygous Notch1 background was recently observed [99], suggesting that cysteine-altering NOTCH3 mutations may not be compulsory to induce GOM. Furthermore, the cysteine-sparing mutation NOTCH3^N3G73A^ promoted aggregation [100], but, in both these cases, the molecular underpinnings for the observed effects need to be explored. Recently, some aspects of CADASIL have been recapitulated in patient-specific induced pluripotent stem cell (iPSC)-derived VSMCs, which fail to stabilize endothelium *in vitro* [101].

One may also ask whether cysteine-sparing NOTCH3 mutations, irrespective of their ability to generate GOM or not, can give rise to neurological or vascular problems simply by altering the signalling output. Two cysteine-sparing NOTCH3 missense mutations with aberrant signalling output (NOTCH3^A1604^T and NOTCH3^L1515P^) have been identified in patients with cerebral small vessel disease and white matter lesions [102,103] (figure 5). The NOTCH3^A1604^T mutation correlated with migraine and white matter lesions in a family, although of a different kind from that which is typical for CADASIL, and was hypomorphic with regard to signalling [103]. The NOTCH3^L1515P^ mutation, in contrast, resulted in hyperactive Notch signalling and correlated with white matter hyperintensities and headache [102]. The prevalence of cysteine-sparing NOTCH3 mutations is not known, and it would be interesting to explore their frequency in larger patient cohorts with broader indications of headache and vascular and neurological problems. A conundrum is that not all cysteine-sparing NOTCH3 mutations appear to give rise to vascular disease; a family carrying a NOTCH3^L1519P^ mutation (located four amino acid residues away from the NOTCH3^L1515P^ mutation) instead presented with infantile myofibromatosis (see above), although the NOTCH3^L1519P^ receptor, like the NOTCH3^L1515P^ receptor, produced hyperactive Notch signalling [70,104].

In addition to cysteine-sparing and -altering NOTCH3 mutations, patients with homozygous nonsense mutations have recently been identified. Homozygous NOTCH3 deficiency was first described in a patient with childhood-onset arteriopathy with white matter abnormalities and microbleeds [105]. Recently, homozygous nonsense NOTCH3 mutations were identified in two patients with Snedden syndrome, a very rare disease that causes ischaemic strokes [106]. Snedden syndrome is, however, also characterized by livedo reticularis (mottled discoloration of the skin), which is not observed in CADASIL, and this underscores that the consequences of complete loss of NOTCH3 function versus missense mutations in the NOTCH3 receptor may be different. It will also be interesting to further explore whether an earlier onset of the ischaemic strokes in the three NOTCH3 null patients identified thus far, as compared with CADASIL patients, is a consistent feature and may reflect more dramatic vascular aberrations.

Pulmonary arterial hypertension (PAH) has been linked to dysfunctional Notch signalling (for a recent review see [79]. PAH is a life-threatening disease, characterized by a persistent elevation of pulmonary arterial pressure caused by pulmonary artery remodelling, thickening of vascular media and luminal occlusion. In PAH, excessive proliferation, anti-apoptosis and dedifferentiation of VSMCs (also known as pulmonary artery smooth muscle cells) are observed. In an important study, Thistlethwaite and colleagues demonstrated that NOTCH3 was upregulated in patients with PAH and that Notch3^−/−^ mice were resistant to developing pulmonary hypertension when exposed to low oxygen levels [107]. The involvement of NOTCH3 in PAH has been substantiated by subsequent studies revealing an upregulation of Notch3 in various PAH animal models [108–111]. Furthermore, the NOTCH3–PAH link has recently been corroborated by the identification of NOTCH3 missense mutations (NOTCH3^G840E^ and NOTCH3^T900P^) in patients with childhood PAH [112] and a NOTCH3 single nucleotide polymorphism (SNP) segregating with childhood PAH [113]. At the endothelial side, NOTCH1 may also play a role in PAH, as NOTCH1 was upregulated in patients with PAH as well as in an experimental PAH rat model, and elevated NOTCH1 levels induced proliferation in lung ECs [114]. There are, however, also observations that loss of Notch1 may aggravate PAH in animal models [115], suggesting that further analyses of the role of Notch1 need to be undertaken.

Aberrant Notch signalling has also been associated with diseases affecting the vasculature of the heart. Patent ductus arteriosus (PDA) is a disease in which the ductus arteriosus fails to close after birth. The VSMC-specific elimination of Jagged1 [116] or the combined loss of Notch3 and specific ablation of Notch2 in VSMCs [117] results in a PDA-like phenotype in mice. Furthermore, NOTCH1 gene variants were observed in patients with aortic coarctation and aortic aneurysm (for a review see [118]), and aberrant expression of Notch pathway genes was noted in patients with thoracic aortic aneurysm [119,120].

In addition to CADASIL, PAH and cardiovascular diseases, the list of diseases with a vascular component and potential links to Notch dysregulation keeps growing (figure 5). Gene fusions between NOTCH1, 2 or 3 with the MIR143 locus were identified in almost 50% of glomus tumours, which constitute a subset of pericytic tumours [121,122]. There is also a potential link between aberrant Notch signalling and arteriovenous malformations (AVMs), a condition in which there is a direct coupling between the arterial and venous vasculature bypassing the capillaries. There are, however, still somewhat conflicting views as to the nature of Notch dysfunction in AVMs. NOTCH1 and NOTCH4 receptor levels are elevated in patients with brain AVMs [123], while in a mouse model reduction of Notch signalling by pericyte-specific targeting of CSL led to AVMs [124]. Specific SNPs for the NOTCH4 gene appear to be associated with AVMs [125], but further research is required to settle whether Notch signalling is hyper- or hypoactivated in this disease. Cerebral cavernous malformation (CCM), which leads to thin-walled vascular cavities and haemorrhages in the brain, is caused by loss-of-function mutations in the CCM1–3 genes [126], and the loss of CCM function is accompanied by reduced Notch signalling and a disruption of Notch signalling between ECs and pericytes [127,128]. Neural tissue haemangioblastoma is characterized by pathological vessel remodelling and can be caused by a mutation in the von Hippel–Lindau (VHL) gene, which encodes an E3 ubiquitin ligase regulating the cellular hypoxic response. It was recently observed in experimental mouse models that VHL mutations impact on Notch signalling, and, importantly, that experimental inhibition of Notch signalling restored some of the effects caused by VHL mutations [129]. A recent study provided evidence that acute blood vessel regeneration following experimental stroke in zebrafish was mediated by transdifferentiation of lymphatic vessels in a Notch-dependent manner [130]. While these examples illustrate several recently discovered links between Notch dysregulation and diseases affecting the vasculature in different organs, there may also be other diseases worth exploring from a Notch vascular perspective. Alagille syndrome is a multi-organ disease presenting with severe liver and heart defects, but also with problems in other organs, such as the eyes and the inner ear. Alagille syndrome is in more than 90% of cases caused by JAGGED1 mutations [131,132], leading to hypomorphic Notch signalling [133]. A significant fraction of patients with Alagille syndrome (up to 10%), however, also experience vascular problems and bleeding [134]. It would be of interest to explore this aspect of the disease and its relation to reduced Notch signalling further, as bleeding in patients with Alagille syndrome remains a major cause of death. Adams–Oliver syndrome is another multi-organ disease with a vascular component; it is caused by loss-of-function mutations in several genes, including NOTCH1, DLL4 and CSL (RBPJ) [135–137]. In addition, mutations are observed in the EGF domain-specific O-linked *N*-acetylglucosamine transferase gene (EOGT), which encodes an enzyme that post-translationally modifies Notch receptors [138]. Patients with Adams–Oliver syndrome show limb malformations and partial absence of skin and skull bones. Furthermore, some patients exhibit vascular problems, including dilated vessels at the surface of the body and pulmonary or portal hypertension. Potential links between aberrant Notch signalling and Alzheimer's disease similarly largely remain to be explored. A recent study proposes that the non-productive angiogenesis observed near Abeta peptide-containing plaques (see below) in Alzheimer's brains may be related to reduced Notch activity [139].

## 7. Notch therapy considerations

With the increasing number of diseases linked to aberrant Notch signalling, there is an obvious need to develop Notch-based therapies, to activate or dampen Notch signalling, depending on the disease situation at hand. Notch therapy development has been vigorously pursued for many years, and although interesting and promising preclinical data are mounting, there are still no Notch-specific therapies in regular clinical use [140,141]. In the category of pan-Notch inhibitors, γ-secretase inhibitors (GSIs), which block the Notch receptor S3 cleavage, have the longest research track record. GSIs were originally developed to block amyloid precursor protein cleavage to generate the Abeta peptide, which aggregates in Alzheimer's disease, but Notch toxicity (including diarrhoea from goblet cell metaplasia and immunological problems) was a severe side effect observed after long-term use in Alzheimer's research clinical trials [140,141]. While this has discouraged interest in GSIs for clinical use in the Alzheimer's field, there is continued interest in exploring their potential use as Notch inhibitors, for example as therapies to Notch-driven cancers. This may potentially be achieved by defining ‘drug holiday' schemes for GSIs such as AL101 and nirogacestat in clinical trials, or in using GSIs in combinatorial therapies with other proven chemotherapy agents [141]. Although all GSIs by definition affect S3 cleavage, it has, however, been proposed that they differ both in efficacy on different Notch receptors and in how they modulate cancer stem cell activity [142]. The differential effect on Notch receptor paralogues is intriguing in light of the fact that their mode of action may be assumed to be quite ‘stereotypical', i.e. affecting S3 cleavage, and it will be interesting to further explore the basis for the potential receptor paralogue specificity for different GSIs. Recently, small-molecule inhibitors that perturb Notch signalling at the level of the ternary transcriptional Notch ICD–MAML–CSL complex [143,144] or at the level of receptor maturation [145] have been developed and it will be interesting to explore their efficacy in modulating Notch signalling in the vasculature. Notch receptor- or ligand-specific blocking antibodies constitute another interesting Notch modulation avenue. Antibodies that block ligand–receptor interaction or lock the NRR in a non-S2-cleavable state have been developed [146,147]. Targeted therapies against Dll4 may be particularly interesting from a tumour vasculature perspective [148], as blockade of Dll4 causes hypersprouting in the vasculature by altering the tip/stalk cell balance, which leads to a poorer blood supply for the tumours.

In addition to Notch inhibitors, there will also be a need for agents that can enhance Notch signalling, for use in situations where endogenous Notch signalling is hypoactive. As discussed above, some CADASIL mutations may fall in this category, and an activating Notch3 antibody was indeed used in a CADASIL mouse model to restore some of the observed phenotypes [98]. It should, however, be kept in mind that some Notch mutations may be refractory to antibody-based strategies aiming at blocking Notch receptor–ligand interaction or the NRR in the extracellular milieu. It was recently shown that the NOTCH3^L1515P^ and NOTCH3^L1519P^ mutations, which cause cerebral small vessel disease and infantile myofibromatosis, respectively, generate mutated hyperactive receptors, which, however, do not reach the cell surface [70,102]; thus, they would not be inhibited by antibody-based approaches. Immunization strategies aiming at targeting NOTCH3-containing aggregates have also been considered for CADASIL therapy [149], and one could envisage active as well as passive immunization strategies for this disease, as the aggregates are extracellular. If immunization were to work, it will be interesting to see if the immunization approach can be generalized using only one form of the mutated NOTCH3 receptor as an antigen, as there are more than 200 different CADASIL mutations in NOTCH3, and it would be impractical and very expensive to tailor immunization to all the different mutations. Finally, Notch receptors, owing to their modular structure and proteolytic processing, can also be used as platforms for novel therapeutics, and synthetic Notch receptors have been developed [150] and used to treat tumours [151].

## 8. Conclusion and future directions

Notch signalling plays a central role in both the development and homeostasis of the vasculature and regulates ECs as well as mural cells. The effects of Notch signalling range from balancing sprouting and branching of the vasculature via regulation of the tip/stalk balance in ECs to control arterial versus venous differentiation of mural cells and the balance between contractile and synthetic phenotypes in homeostasis, stress and disease. The number of vascular diseases that are caused or influenced by dysregulated Notch signalling is increasing and warrants efforts of developing Notch-based therapies. Despite recent progress, there are, however, several outstanding questions that remain to be answered, including how Notch produces appropriate downstream molecular outputs in the different cell types, given the relatively simple architecture of the pathway. Progress in this area is anticipated, not least because of rapid technological improvements and scalability of single-cell transcriptomics combined with increasingly precise transgenic models in both mouse and zebrafish. Detailed transcriptomic maps are beginning to be produced of the vasculature in different organs [28,152–154], and will be complemented with analyses from corresponding maps from various disease conditions. We also expect progress in understanding how the Notch signalling is modulated by auxiliary proteins in the pathway, including glycosylation enzymes, kinases and phosphatases, which in various ways modulate the signalling output. Such endeavours will also be important for the development of novel concepts for Notch-based therapeutics.

## Data Availability

This article has no additional data.
